# Ocular surface disease: a known yet overlooked side effect of topical glaucoma therapy

**DOI:** 10.3389/ftox.2023.1067942

**Published:** 2023-07-21

**Authors:** Raul E. Ruiz-Lozano, Nadim S. Azar, Hazem M. Mousa, Manuel E. Quiroga-Garza, Seitaro Komai, Lorena Wheelock-Gutierrez, Cristian Cartes, Victor L. Perez

**Affiliations:** ^1^ Tecnologico de Monterrey, Escuela de Medicina y Ciencias de La Salud, Monterrey, Mexico; ^2^ Department of Ophthalmology, Foster Center for Ocular Immunology at Duke Eye Center, Duke University School of Medicine, Durham, NC, United States; ^3^ Asociación Para Evitar La Ceguera en México, I.A.P, Mexico City, Mexico; ^4^ Unidad Oftalmología, Departamento de Especialidades, Facultad de Medicina, Universidad de La Frontera, Temuco, Chile

**Keywords:** alpha-adrenergic agonists, beta blockers, carbonic anhydrase inhibitors, dry eye disease, nitric oxide-donating prostaglandin analogs, ocular surface disease, prostaglandin analogs, rho-kinase inhibitors

## Abstract

Ocular surface disease (OSD), a disorder affecting the lacrimal and meibomian glands and the corneal and conjunctival epithelium, is a well-known complication of topical glaucoma therapy. OSD can present as a new or pre-existing condition that virtually any anti-glaucoma formulation can exacerbate. As such, both glaucoma and OSD frequently coexist. Typical OSD symptoms include ocular discomfort, redness, burning, and dryness, whereas signs include periorbital and eyelid skin pigmentation, conjunctival scarring, and superficial punctate keratitis. Pressure-lowering eyedrops can cause toxic, allergic, and inflammatory reactions on the ocular surface. The latter can result from either preservatives or direct toxicity from the active molecule. Although usually mild, OSD can cause significant symptoms that lead to poor quality of life, decreased compliance to therapy, glaucoma progression, and worse visual outcomes. Given the chronic nature of glaucoma, lack of curative therapy, and subsequent lifelong treatment, addressing OSD is necessary. This manuscript aims to provide an up-to-date overview of OSD’s signs, symptoms, and pathogenic mechanisms from glaucoma therapy toxicity.

## 1 Introduction

Glaucoma is a group of diseases leading to progressive optic neuropathy, characterized by visual field and optic nerve head changes (Aguayo Bonniard et al., 2016). It is the primary cause of irreversible blindness worldwide, with a prevalence ranging from 2.0% in Europeans to 7.3% among individuals of African descent (Sun et al., 2022). Unfortunately, the lack of a cure renders glaucoma therapy lifelong (Aguayo Bonniard et al., 2016). Moreover, disease progression requiring more than one anti-glaucomatous agent occurs in approximately 40% of glaucoma patients. The latter results in chronic exposure and toxicity to the active molecules and associated preservatives (Anwar et al., 2013).

Ocular surface disease (OSD) represents a spectrum of diseases, including conjunctivitis, lid disease, allergic manifestations, superficial punctate keratitis, and dry eye disease (DED) (Fechtner et al., 2010; Saade et al., 2015; Gomes et al., 2017). Both glaucoma and OSD are prevalent in the elderly and frequently coexist in the same patient (Fechtner et al., 2010). Up to 66% of patients with severe OSD have glaucoma (Fechtner et al., 2010), whereas the prevalence of OSD in patients using topical anti-glaucoma agents is as high as 59% (Zhang et al., 2019). Patients with prior DED, those exposed to preserved pressure-lowering medications (PLMs), and those requiring ≥1 agent (Saade et al., 2015), are at an increased risk of experiencing worse OSD symptoms. Furthermore, dry eye symptoms can result in increased patient depression, anxiety, and poor quality of life (QoL), which, in turn, is associated with poor compliance to glaucoma therapy and an increased risk of glaucoma progression (Stringham et al., 2018; Tirpack et al., 2019; Rodriguez-Garcia et al., 2022). Thus, addressing OSD in patients with glaucoma is necessary.

This review discusses the pathogenic mechanisms and diagnosis of ocular surface toxicity induced by anti-glaucoma agents, with emphasis on the newer drugs: the rho-kinase (ROCK) inhibitors and nitric oxide (NO)-donating prostaglandin analogs (PGAs).

## 2 Mechanism of action of pressure-lowering medications

The mechanism of action of PLMs can be divided into those decreasing the aqueous humor production and those increasing the trabecular meshwork or uveoscleral outflow. Regarding the latter, PGAs are the first-line PLM for managing ocular hypertension (OHT) and glaucoma. They exert their effects by binding to E prostanoid and F prostanoid receptors, leading to ciliary muscle relaxation and increased aqueous humor outflow through the uveoscleral pathway (Yamagishi-Kimura et al., 2022). PGAs also induce the expression of matrix metalloproteinases (MMPs) that disrupt the extracellular matrix (ECM), leading to increased trabecular meshwork outflow. Hence, PGAs lower intraocular pressure (IOP) by increasing flow through both pathways (Heo et al., 2020).

Table 1 describes the mechanism of action of each PLM class. However, in the following subsections, we will discuss the mechanism of action of the newer classes of PLMs: the ROCK inhibitors and the NO-donating PGAs.

**TABLE 1:** Mechanisms of action of pressure-lowering medications.

Drug class	Mechanisms of action	Examples	References
**Increased uveoscleral and trabecular meshwork outflow**			
PGAs	- Causes relaxation of the ciliary muscle by binding to the FP and EP-1, -2, -3, and -4 receptors causes relaxation of the ciliary muscle	Latanoprost, bimatoprost, unoprostone, travoprost, and tafluprost	Nilsson et al. (2006); Alm and Nilsson. (2009); Agarwal and Agarwal. (2018); Karli et al. (2018)
	- Degrades the ECM by activating MMPs-1, -2, -3, and -9 in ciliary body smooth muscle cells		
**Decrease aqueous humor production**			
Beta blockers	- Antagonize the effects of catecholamines on β2-adrenoreceptors in the ciliary epithelium	*Cardioselective*: betaxolol	Nyborg and Nielsen. (1995); Kiland et al. (2004); Servat and Bernardino. (2011)
	- Vasoconstriction of ciliary arteries	*Non-selective*: timolol, carteolol, levubonolol, and metipranolol	
CAIs	- Inhibit carbonic anhydrase isoenzymes present in the ciliary processes	Dorzolamide and brinzolamide	Sugrue et al. (1990); Mincione et al. (2007); Shahidullah et al. (2009)
	- Inhibition of HCO_3_^−^ and CO_2_ interconversion		
Alpha agonists	- Activation of α1- and α2-adrenoreceptors inhibits adenylate cyclase, causing a decrease in cAMP.	Apraclonidine and brimonidine	Bausher and Horio, (1995); Servat and Bernardino, (2011)
**Increased trabecular meshwork outflow**			
Cholinergics	- Acts on iris muscles’ muscarinic receptors, causing pupillary and ciliary muscle contraction, decreasing resistance to aqueous humor outflow	Pilocarpine, bethanecol, and carbachol	Kam and Sullivan, (2011); Zhang et al. (2017)
**Mixed mechanisms**			
ROCK inhibitors	- Inhibits ROCK and NE transporters in the trabecular pathway, which reduces aqueous humor production, increases trabecular outflow, and decreases EVP.	Netarsudil, fasudil, and ripasudil	Serle et al. (2018); Batra et al. (2021)
NO-donating PGA	- LBN is metabolized into butanediol mononitrate, a NO-donating moiety, and latanoprost acid (increases uveoscleral outflow)	Latanoprostene bunod	Lo et al. (2022)
	- NO increases TM outflow via TM and Schlemm’s canal relaxation		

PGAs, prostaglandin analogs; FP, F prostanoid; EP, E prostanoid; ECM, extracellular matrix; MMPs, matrix metalloproteinases; CAIs, carbonic anhydrase inhibitors; HCO_3_^−^, bicarbonate; CO_2_, carbon dioxide; cAMP, cyclic adenosine monophosphate; ROCK, rho-kinase; NE, norepinephrine; EVP, episcleral venous pressure; NO, nitric oxide; LBN, latanoprostene bunod; TM, trabecular meshwork.

### 2.1 Rho-kinase (ROCK) inhibitors

Rho-associated coiled-coil-containing protein kinase (ROCK) is the most studied downstream effector of RhoA, a guanosine triphosphate (GTP)-ase member of the Rho subfamily of the Ras protein family. Rho is activated in the GTP-binding state. The process is aided by bioactive receptors like endothelin-1 and transforming growth factor (TGF)-β, which activates GTPase activating proteins (GAP) and guanine nucleotide exchange factors (GEFs), leading to Rho activation through GTP-binding (Berrino and Supuran, 2019).

ROCKs exhibit two isoforms (ROCK-I and ROCK-II) expressed in many body organs, with varying extents of the subtype involved in each organ and cell tissue. Within the eye, ROCKs are expressed in the trabecular meshwork. In a calcium-independent manner, ROCKs contract the trabecular meshwork by phosphorylating LIM kinases and myosin light chain (MLC) phosphatase. This creates resistance to aqueous humor outflow in the trabecular meshwork (Berrino and Supuran, 2019; Bhargava et al., 2022). ROCK inhibitors decrease actin fiber density in the trabecular meshwork and increase endothelial cell permeability in Schlemm’s canal, ultimately counteracting this outflow resistance (Moura-Coelho et al., 2019).

Netarsudil, a specific ROCK inhibitor, has an amplified impact as it is also a norepinephrine transporter (NET) inhibitor that further decreases aqueous humor production and venous pressure in episcleral vessels (Bhargava et al., 2022).

### 2.2 Nitric oxide (NO)-donating prostaglandin analogs (PGAs)

Under physiologic conditions, NO is expressed by trabecular meshwork cells, Schlemm’s canal, and the ciliary body. NO reduces IOP by increasing aqueous humor outflow from the trabecular meshwork by activating the soluble guanylate cyclase (sGC)-cyclic guanosine monophosphate (cGMP) pathway and by decreasing aqueous humor production through ion channel modulation. cGMP regulates the action of various downstream effectors, including protein kinase G (PKG), that relaxes vascular smooth muscle (Mao et al., 2020). Via the NO/sGC/cGMP pathway, PKG activates MLC phosphatase, which, in turn, dephosphorylates MLC, leading to the relaxation of trabecular meshwork and Schlemm’s canal cells. The hindmost decreases aqueous humor outflow resistance (Gao et al., 2017). In addition, inducible NO synthase can be activated in trabecular meshwork cells when anterior chamber perfusion pressure becomes elevated (Wu and Ma, 2012).

Latanoprostene bunod is a NO-donating prostaglandin F2α analog recently approved by the FDA (2017) for managing glaucoma (Krauss et al., 2011). When topically administered, the compound is split into the conventional drug and NO, potentiating the IOP-lowering effect (Krauss et al., 2011; Mao et al., 2020). Thus, NO-donating PGAs offer two mechanisms of action. The NO component promotes outflow through the conventional pathway, and the prostaglandin component facilitates flow through the uveoscleral pathway space (Cavet et al., 2015).

## 3 General mechanisms of ocular surface toxicity caused by pressure-lowering medications

The lacrimal functional unit (LFU) is comprised of the eyelids, meibomian glands (MGs), the main and accessory lacrimal glands, the lacrimal drainage system, the ocular surface (cornea and conjunctiva), and the intertwined innervation (Baudouin et al., 2013). A healthy ocular surface relies on the LFU, which is responsible for the adequate production, distribution, and clearance of the tear film. The latter, in turn, preserves homeostasis of the ocular surface epithelium and protects it from physical damage and exposure (Kawakita, 2018). Dysfunction of one or more components of the LFU 1) hinders the composition of tears, and thus its ability to protect the surface epithelium; 2) disrupts the innate and adaptive immune and inflammatory pathways that protect the ocular surface from external stimuli (i.e., exposure, infection); and 3) stimulate the production of proinflammatory cytokines [i.e., interferon (IFN)-γ, interleukin (IL)-1, −6, −8, tumor necrosis factor (TNF)-α, intercellular adhesion molecule (ICAM)-1, among others] by the ocular surface immune and epithelial cells (Roy et al., 2022).

**FIGURE 1:**
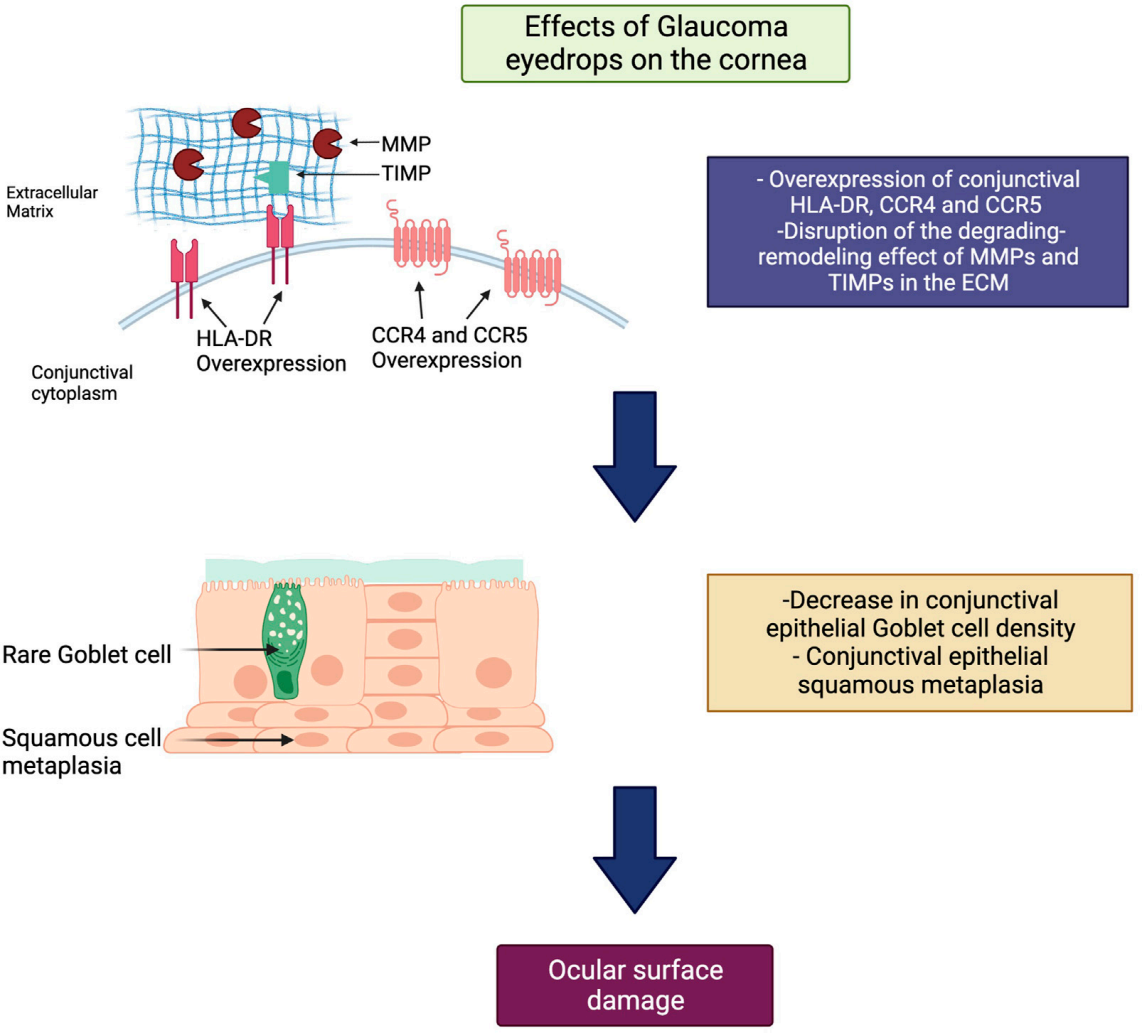
General mechanism of ocular surface toxicity due to pressure-lowering medications (PLMs). Within the ocular surface, PLMs cause overexpression of C-C chemokine receptors (CCR)-type 4 and 5, leading to an IgE-mediated allergic reaction on the ocular surface. Also, they cause to disrupt the degrading-remodeling balance between matrix metalloproteinases (MMPs) and tissue inhibitors of metalloproteinases (TIMPs) in the extracellular matrix (ECM). The latter results in decreased goblet cell density, squamous metaplasia, and ocular surface damage. Footnote: HLA-DR, human leukocyte antigen DR isotype; MMPs, matrix metalloproteinases; TIMPs, tissue inhibitors of metalloproteinases; ECM, extracellular matrix; CCR, C-C chemokine receptor.

**FIGURE 2:**
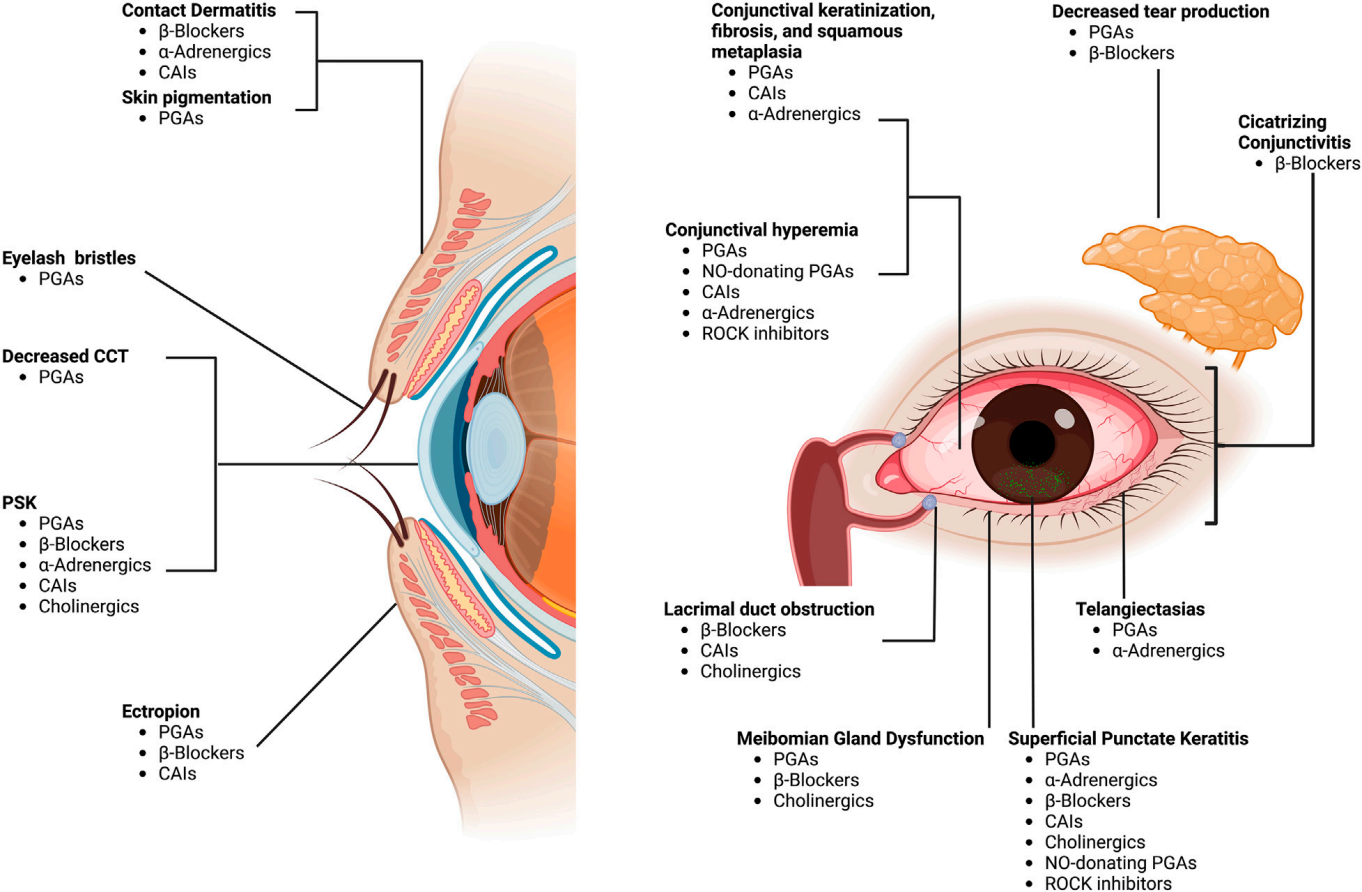
Ocular surface adverse effects of pressure-lowering medications. Footnote: CAIs, carbonic anhydrase inhibitors; PGAs, prostaglandin analogs; CCT, central corneal thickness; PSK, pseudodendritic sterile keratitis; NO, nitric oxide; ROCK, rho-kinase. “Created with BioRender.com”.

In this regard, PLMs for the management of OHT and glaucoma have been shown to cause damage to the LFU through a myriad of mechanisms, including a decrease in conjunctival goblet cell (GC) density, squamous metaplasia (Baudouin et al., 2008), conjunctival human leukocyte antigen (HLA)-DR overexpression (Baudouin et al., 2008), and disruption of the degrading-remodeling effect between MMPs and tissue inhibitors of metalloproteinases (TIMPs) in the ECM compounds, including collagen fibers (Karli et al., 2018). Moreover, the need for fixed combinations, commonly required by patients exhibiting disease progression and lifelong treatment, as well as the vehicles and preservatives contained in drug formulations [i.e., benzalkonium chloride (BAK)], will result in a significant number of patients experiencing ocular surface damage (Tiedemann et al., 2019; Rodriguez-Garcia et al., 2022). Additionally, an impression cytology study reported significant overexpression of C-C chemokine receptors (CCR)-type 4 and 5 in the conjunctival epithelium of glaucoma subjects chronically treated with PLMs compared with controls (Baudouin et al., 2008). CCR4 is expressed by the Th2 pathway, which is involved in IgE-mediated allergic diseases, whereas CCR5 is expressed by the Th1 pathway, which has a role in type IV hypersensitivity reactions and the immune response to infections (Baudouin et al., 2005) (Figure 1). These findings suggest that, aside from the inflammatory and toxic mechanisms, allergy may also play a role in the ocular surface damage experienced by glaucoma patients treated with PLMs. Table 2 and Table 3 present the ocular surface disease manifestations of PLMs preserved with BAK and other additives, respectively (Figure 2).

**TABLE 2:** Human studies reporting ocular surface disease manifestations of BAK-containing pressure-lowering medications.

Drug class (frequency)		Periocular changes	Lacrimal drainage system	Meibomian glands	Conjunctiva	Cornea	References
PGAs	Frequent	Eyelash bristles	Epiphora	MG atrophy	Hyperemia	SPK	Inoue et al. (2012b); Lopilly Park et al. (2012); Sakata et al. (2013); Peace et al. (2015); El Hajj Moussa et al. (2018); Staso et al. (2018); Chang et al. (2021); Singh et al. (2022); Di Maria et al. (2023)
		Skin pigmentation		MG dropout	SQ metaplasia	↓CCT	
				↓TF stability	↓GCD	↑DCC	
				↓MAD		Irregular LTE	
				↓MAA		↑Nerve tortuosity	
						↓ECD	
	Rare	DUES	Fibrosis	None	DICC	PSK	
		Periocular CD	NLDO				
		Periocular ACD					
Beta blockers	Frequent	Periocular CD	↓Schirmer	MG atrophy	Hyperemia	SPK	Jappe et al. (2006); Hegde et al. (2007); Servat and Bernardino, (2011); Frezzotti et al. (2014); Ortiz-Basso et al. (2018); Staso et al. (2018); Kim et al. (2021b); Chang et al. (2021); Singh et al. (2022)
		Periocular ACD		MG dropout	SQ metaplasia	↑DCC	
				↓TF stability	↓GCD	Irregular LTE	
						↓ECD	
	Rare	DI-ectropion	NLDO	None	DICC	PSK	
CAIs	Frequent	Periocular ACD	Epiphora	MG atrophy	Hyperemia	SPK	Delaney et al. (2002); Hong et al. (2006); Hegde et al. (2007); Servat and Bernardino, (2011); Ortiz-Basso et al. (2019); Chang et al. (2021); Singh et al. (2022)
				MG dropout	Allergy		
				↓TF stability	SQ metaplasia		
	Rare	DI-ectropion	NLDO	None	DICC	PSK	
						Corneal failure	
Alpha agonists	Frequent	Periocular ACD	Epiphora	MG atrophy	Hyperemia	SPK	Servat and Bernardino, (2011); Aydin Kurna et al. (2014); Ortiz-Basso et al. (2018); Duru and Ozsaygili, (2020); Chang et al. (2021); Singh et al. (2022)
				MG dropout	Allergy		
				↓TF stability			
	Rare	Periocular CD	NLDO	None	Edema	None	
		DI-ectropion					
Miotics	Frequent	Periocular CD	None	MG atrophy	Hyperemia	SPK	Servat and Bernardino, (2011); Grey and Warshaw, (2016); Zhang et al. (2017); Ortiz-Basso et al. (2018); Chang et al. (2021); Singh et al. (2022)
				MG dropout	SQ metaplasia	Haze	
				↓TF stability			
	Rare	Periocular ACD	NLDO	None	DICC	PSK	
ROCK inhibitors	Frequent	Eyelid erythema	Epiphora	None	Hyperemia	SPK	Wisely et al. (2020); Kim et al. (2021a); Batra et al. (2021); Meirick et al. (2022)
					Hemorrhage	Cornea verticillata	
	Rare	Eyelid wound dehiscence	Transient punctal stenosis	None	None	Reticular bullous epithelial edema	
NO- donating PGAs	Frequent	Skin pigmentation	None	None	Hyperemia	SPK	Kawase et al. (2016); Lo et al. (2022)
	Rare	None	None	None	None	None	

BAK, benzalkonium chloride; PGAs, prostaglandin analogs; MG, Meibomian gland; TF, tear film; MAD, mean acinar density; MAA, mean acinar area; SQ, squamous; GCD, goblet cell density; SPK, superficial punctate keratitis; CCT, central corneal thickness; DC, dendritic cell density; LTE, limbal transition epithelium; ECD, endothelial cell density; DUES, deepening upper eyelid sulcus; CD, contact dermatitis; ACD, allergic contact dermatitis; NLDO, nasolacrimal duct obstruction; DICC, drug-induced cicatrizing conjunctivitis; PSK, pseudo-dendritic keratitis; DI, drug-induced; CAIs, carbonic anhydrase inhibitors; ROCK, rho kinase; NO, nitric oxide.

**TABLE 3:** Human studies reporting ocular surface disease manifestations of non-BAK preserved pressure lowering medications.*^†^

Drug class	Findings	Polyquad	Sofzia	Purite	References
**PGAs**	**Periocular changes**	Skin pigmentation	Skin pigmentation	-	Kammer et al. (2010); Mizoue et al. (2014); Lopes et al. (2015); Peace et al. (2015); El Hajj Moussa et al. (2018); Ortiz-Basso et al. (2018); Muz et al. (2021)
			DUES		
	**LD system**	↓Schirmer	Not reported	-	
		↓tear film stability			
	**Meibomian glands**	MG dropout	MG dropout	-	
		↓tear film stability	↓tear film stability		
	**Conjunctiva**	Hyperemia	Hyperemia	-	
		Edema			
	**Cornea**	Punctate keratitis	Punctate keratitis	-	
		↓Corneal hysteresis			
**Beta blockers**	**Periocular changes**	Not reported^‡^	-	-	Schnober et al. (2015)
	**LD system**	Not reported^‡^	-	-	
	**Meibomian glands**	Not reported^‡^	-	-	
	**Conjunctiva**	Hyperemia^‡^	-	-	
	**Cornea**	Punctate keratitis^‡^	-	-	
**Alpha agonists**	**Periocular changes**	-	-	Eyelid edema	Whitson et al. (2006); Duru and Ozsaygili. (2020)
				Periocular CD	
	**LD system**	-	-	↓Schirmer	
	**Meibomian glands**	-	-	Not reported	
	**Conjunctiva**	Hyperemia	-	Hyperemia	
		Allergic conjunctivitis		Allergic conjunctivitis	
				↓Goblet cell density	
	**Cornea**	-	-	Punctate keratitis	
				PSK	

*Blank cells indicate that there are no available formulations containing of the drug class and the preservative.

^†^There are no available formulations of Polyquad-, Sofzia, and Purite-preserved carbonic anhydrase inhibitors, miotics, rho kinase inhibitors, and nitric-oxide donating PGAs.

^‡^Polyquad-preserved beta blockers only exist in fixed combinations with prostaglandin analogs (travoprost).

*BAK, benzalkonium chloride; PGAs, prostaglandin analogs; DUES, deepening upper eyelid sulcus; LD,* lacrimal *drainage; MG, Meibomian gland; CD, contact dermatitis; PSK, pseudo-dendritic keratitis.*

## 4 Specific ocular surface disease changes caused by pressure-lowering medications

### 4.1 β-Adrenergic antagonists (β-blockers)

#### 4.1.1 Contact dermatitis

**FIGURE 3:**
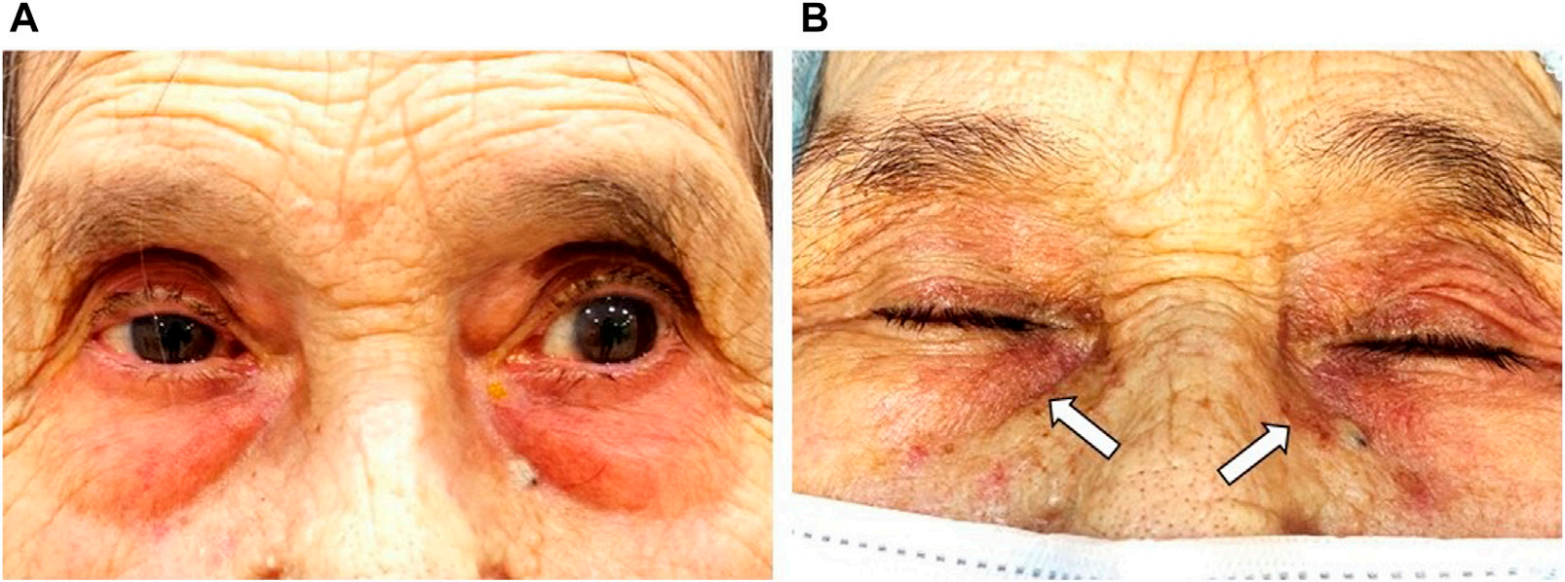
A 91-year-old female patient with a history of chronic angle-closure glaucoma treated with benzalkonium chloride (BAK)-containing 0.03% bimatoprost qHs and a fixed combination of 0.5% timolol maleate/2% dorzolamide BID eyedrops for at least 10 years. **(A)** Significant erythema of the periorbital skin and lid margin are consistent with allergic contact dermatitis. **(B)** After 4 weeks of treatment with ocular lubricants and preservative-free (PF) IOP-lowering eyedrops, there was a significant reduction of the periorbital skin and lid margin erythema. However, residual skin pigmentation (white arrows) is observed. Footnote: Written informed consent was obtained from both patients to publish the clinical images.

Periocular contact dermatitis is a frequent adverse effect of topical anti-glaucomatous agents, mainly β-blockers (Horcajada-Reales et al., 2015) (Figure 3A). It may present as erythema with or without eczema and crusting of the eyelids. Koch et al. reported sensitization to a single β-blocker despite previous exposure to other β-blockers in three patients (Koch, 1995). This was also reported by Perez-Rodriguez et al. in a patient sensitized to 0.005% latanoprost but not to 0.03% bimatoprost (Perez-Rodriguez et al., 2008) and by Geyer et al. in 15 patients with proven allergy to 0.5% apraclonidine but without cross-reactivity to 0.25% clonidine hydrochloride (Geyer et al., 2000). Contrariwise, other authors report positive patch testing for multiple β-blockers, suggesting cross-sensitization (Horcajada-Reales et al., 2015). While some authors suggest cross-reactivity between multiple β-blockers might result from a common lateral aliphatic chain acting as an antigenic determinant others hypothesize that positive reactions could be related to multiple sensitizations instead of cross-reactivity. Allergic contact dermatitis in patients naïve to other PLMs from the same or other group has been reported with dorzolamide, brimonidine tartrate, and cholinergic agonists (i.e., pilocarpine) (Grey and Warshaw, 2016).

Interestingly, other studies report the development of a clinically apparent allergic reaction but with negative patch testing. Giordano-Labadie et al. reported the case of a patient who developed negative-patch chronic eczema for timolol, carteolol, and befunolol. The authors suggested cross-reactivity occurred after drugs were metabolized to a common aldehyde rather than a reaction to an individual hapten (Giordano-Labadie et al., 1997). Written informed consent was obtained from all patients to publish the clinical images used throughout the manuscript.

#### 4.1.2 Meibomian gland dysfunction (MGD)

Sullivan and coworkers hypothesized that the drug action of anti-glaucomatous agents might contribute to DED development by a direct effect on MGs (Zhang et al., 2017; Han et al., 2018; Han et al., 2020). They performed a series of experiments in which they cultured immortalized human MG epithelial cells (iHMGEC) with different concentrations of several α-adrenergic agonists (brimonidine, clonidine, phenylephrine) (Han et al., 2018), dorzolamide (Han et al., 2020), pilocarpine, and timolol (Zhang et al., 2017). Using clinical doses of 0.5% timolol and 4% pilocarpine (See Section 4.5.2), Zhang et al. demonstrated they both caused significant cell atrophy and death of iHMGEC (Zhang et al., 2017). Regarding timolol, MG dropout might be associated with the blockade of β_3_-adrenoreceptors, which has been found in MGs of murine models (Knop et al., 2011). These receptors mediate fat oxidation and increase lipolysis; thus, beta-adrenergic blockade might cause detrimental effects on iHMGEC (Zhang et al., 2017). Arici et al. reported significantly lower TFBUT scores, a surrogate marker of increased evaporation due to MGD, in patients treated with BAK-containing 0.5% betaxolol or 0.5% timolol eyedrops compared with controls (Arici et al., 2000). Table 4 presents the recent relevant *ex-vivo* and *in-vitro* human studies reporting OSD manifestations of preserved PLMs.

**TABLE 4:** Recent relevant *ex-vivo* and *in-vitro* human studies reporting ocular surface disease manifestations of PLMs.

	Cell type	Agents used	Study description	Preservatives	Findings
References					
Park et al. (2011)	*Ex-vivo* orbital fat	PGAs	Evaluate the adipocyte density in orbital fat after exposure to preserved PGAs	BAK, PQ	The mean adipocyte density was significantly increased in eyes exposed to preserved PGAs, suggesting adipocyte atrophy
Choi et al. (2012)	Orbital adipocytes	PGAs	Evaluate the effects of PF PGAs in orbital fat	None	LAT, TRV, BIM, and TAF inhibited intracellular lipid accumulation and preadipocyte differentiation
Seibold et al. (2013)	*In vitro* subcutaneous adipocytes	BBs and PGAs	Compare the short-term effects of PF-TIM, various PGAs, and BAK alone on adipocyte cytotoxicity and preadipocyte proliferation	BAK	PF-TIM and BAK alone yielded anti-proliferative effects on pre-adipocytes and cytotoxic effects on mature adipocytes compared with the minimal toxicity caused by PGAs
Lopilly Park et al. (2012)	*Ex-vivo* tears	PGAs and BBs	Proteomic analysis of tears from patients using TIM, or various preserved PGAs, including LAT, TRV, or BIM for >1 year	BAK	Increased levels of MMP-1, MMP-3, MMP-9, IL-1β, IL-6, and decreased levels of TIMP-1 and TIMP-2 in PGA treated eyes compared with TIM
Mohammed et al. (2020)	*Ex-vivo* tears	PGAs	Evaluate the profile of inflammatory cytokines among various preserved and PF PGAs	BAK, PQ	BAK-preserved PGAs induced significant mRNA and protein expression of IL-1β, IL-6, and IL-8 compared with PQ and PF-PGAs
Zhang et al. (2017)	*In vitro*	BBs and miotics	Evaluate the effects of PF TIM and PIL in MG epithelial cells	None	TIM and PIL resulted in dose-dependent atrophy and dropout of MG epithelial cells
	MGs				
Han et al. (2018)	*In vitro*	AAs	Evaluate the effects of various AAs on the structure and function of MG epithelial cells	None	Brimonidine elicits a dose-dependent differentiation of MG epithelial cells, increasing neutral lipidsand lysosome levels
	MGs				
Rath et al. (2019)	*In vitro*	PGAs	Evaluate the effects of various preserved and PF PGAs on MG epithelial cells	BAK, PQ	Cell viability was significantly reduced in BAK-containing PGAs and BAK alone compared with PF PGAs and TRV with PQ
	MGs				
Ammar et al. (2010)	*In vitro* cornea and conjunctiva	PGAs	Percentage of living epithelial cells to different preserved PGAs	BAK, PQ, SZ	PQ and SZ resulted in higher percentages of living cells compared with BAK
Whitson and Petroll (2012)	*In vitro* cornea	PGAs	Evaluate the toxicity of preserved and PF PGAs in the corneal epithelium	BAK, PQ	BAK-containing formulations resulted in significantly greater toxicity and less cell viability
Paimela et al. (2012)	*In vitro* cornea	PGAs	Determine the cytotoxic and inflammatory effects of preserved LAT and TRV	BAK, PQ	PQ-containing TRV activated NF-κB and significantly increased IL-6 and IL-8 compared with BAK
Yuan et al. (2016)	*In vitro* cornea	Miotics	Evaluate the cytotoxic effects of pilocarpine in stromal cells	None	Pilocarpine can induce apoptosis of corneal stromal cells in a dose-dependent manner
Liang et al. (2022)	*In vitro* conjunctiva and cornea	PGAs	Effects of preserved and PF PGAs in a wound-healing epithelial cell model	BAK, PQ, SZ	BAK significantly delayed healing through decreased Ki-67-positive cell numbers and actin disorganization compared to PQ, SZ, and PF-PGAs
Hedengran et al. (2022)	*In vitro* conjunctiva	PGAs	Viability of goblet cells and secretion of cytokines and mucins after exposure to TRV	BAK, PQ	PQ-containing TRV resulted in no goblet cell loss. Both PQ and BAK showed no differences in mucin and IL-6 and IL-8 secretion
Hedengran et al. (2021)	*In vitro* conjunctiva	CAIs, AAs, and miotics	Evaluate the effects of BAK-containing PLMs in conjunctival GCs	BAK	BAK-preserved LAT, followed by DORZ, resulted in significantly less GC density. BRIM did not affect GC survival

PLMs, pressure-lowering medications; PGAs, prostaglandin analogs; BAK, benzalkonium chloride; PQ, polyquad; PF, preservative-free; LAT, latanoprost; TRV, travoprost; BIM, bimatoprost; TAF, tafluprost; BBs, beta blockers; TIM, timolol; MMP, matrix metalloproteinase; TIMP, tissue inhibitor of metalloproteinase; IL, interleukin; MGs; Meibomian glands; AAs, alpha agonists; SZ; Sofzia; NF-κB, nuclear factor kappa beta; GC, goblet cells.

#### 4.1.3 conjunctival goblet cell (GC) dropout

β-blockers cause abnormal keratinization, squamous metaplasia, inflammation leading to GC loss, and subconjunctival fibrosis (Singh et al., 2022). An impression cytology study reported that 50% and 55% of samples treated with BAK-containing 0.5% betaxolol or 0.5% timolol, respectively, were classified as grade 2, defined as the presence of large and multinucleated epithelial cells and a marked reduction of GCs, or grade 3, defined as even larger epithelial cells and complete absence of GCs (Arici et al., 2000). Terai et al. performed a histological analysis of human conjunctiva, evaluating the effect of BAK-containing 0.5% timolol and 0.005% latanoprost on MMPs and TIMPs expression and ECM organization (Terai et al., 2009). Compared with latanoprost, timolol-treated eyes exhibited overexpression of CD68 antibodies, an indicator of inflammatory infiltration. The latter suggests that chronic exposure to timolol eyedrops might result in conjunctival scarring and the potential for filtering surgery (trabeculectomy) failure (Terai et al., 2009).

A study performed by Aydin Kurna and coworkers evaluated the effect of different anti-glaucoma formulations, including preserved and preservative-free (PF)-timolol, and preserved formulations of latanoprost, bimatoprost, travoprost, and brimonidine (Aydin Kurna et al., 2014). At the 12-month follow-up, a significant increase in superior-central and inferior-nasal squamous metaplasia was observed in the brimonidine and both preserved and PF-timolol maleate groups. In PGA-treated eyes, an increase in the inferior-nasal squamous metaplasia was only reported in the BAK-containing travoprost group. Regarding GC loss, significant superior-central and inferior-nasal loss were observed in the PF-timolol and BAK-travoprost groups. In contrast, a consequential inferior-nasal loss was documented in the preserved-latanoprost and -brimonidine groups (Aydin Kurna et al., 2014).

#### 4.1.4 drug-induced cicatrizing conjunctivitis (DICC)

DICC, also known as “pseudo-pemphigoid,” is the development of conjunctival scarring after exposure to an inciting agent (Singh et al., 2022). It may be non-progressive or progressive, depending on whether the scarring process stabilizes (or not) after the withdrawal of the inciting agent (Singh et al., 2022). Although DICC can be associated with any anti-glaucoma medication, β-blockers are, by far, the most frequently reported. In 41 patients with DICC, β-blocker exposure was reported in 36 cases (88%). Timolol maleate was the culprit in 73% of cases (Thorne et al., 2004). The pathogenic mechanism of DICC consists of an inflammatory and immunological process leading to limbal stem cell deficiency, subconjunctival fibrosis, and fornix foreshortening, mainly of the inferior bulbar and palpebral conjunctiva (Vazirani et al., 2020). However, ocular signs may involve the entire surface, including punctum scarring, periocular hypopigmentation, obstructive MGD, eyelash overgrowing (distichiasis), malposition (trichiasis), and lid margin keratinization (Singh et al., 2022).

Histopathological findings of DICC are very similar to those encountered in other cicatrizing conditions such as ocular mucous membrane pemphigoid (OMMP), notably showing increased proliferation of the basal cells of the conjunctival epithelium, marked infiltration of inflammatory cells such as macrophages, neutrophils, and T-lymphocytes in the acute phase, and fibroblast stimulation resulting in fibrosis in the chronic phase (Elder and Lightman, 1994; Singh et al., 2022). However, a notable distinction between DICC and OMMP can be made with direct immunofluorescence (DIF). While DIF following a conjunctival biopsy of a patient with OCP shows linear deposition of IgA, IgG, IgM, and complement C3 on the conjunctival epithelial basement membrane zone, DIF in pseudo-pemphigoid cases such as DICC is usually negative for these observations and require clinical diagnoses (Singh et al., 2022).

Gibran reported the case of an 85-year-old woman with an 8-year BAK-containing latanoprost and apraclonidine use to manage pseudo-exfoliative glaucoma in her left eye (Gibran, 2004). Symptoms included painful red eye and blurred vision, whereas signs included keratoconjunctivitis sicca, fornix foreshortening, and corneal scarring with active neovascularization in the left eye. The right eye was normal (Gibran, 2004). A similar case of a patient exposed to multiple BAK-containing drugs, including latanoprost, dorzolamide, brinzolamide, pilocarpine, and brimonidine, was also reported by Kahana et al. (Kahana et al., 2007). In both cases, BAK was deemed responsible for the development of DICC (Gibran, 2004; Kahana et al., 2007).

#### 4.1.5 Lacrimal drainage obstruction (LDO)

Topical anti-glaucoma agents can cause isolated canalicular and lacrimal occlusion, as well as a more extensive cicatrizing process known as drug-induced cicatrizing conjunctivitis (DICC, See Section 4.1.4) (Kashkouli et al., 2008). Narioka et al. found a decrease in the lumen width of the nasolacrimal drainage (NLD) system after exposure to 0.5% timolol, located mainly in the middle and lower regions (Narioka and Ohashi, 2007). These findings imply that timolol caused vasodilation of the blood vessels in the NLD system’s cavernous body, suggesting that the autonomic nervous system may partially control tear drainage through the NLD system (Narioka and Ohashi, 2007). In a large prospective and controlled case series of 627 eyes from 384 patients, Kashkouli et al. reported significant lacrimal drainage obstruction (LDO) in patients using combined formulations of timolol/dorzolamide and timolol/dorzolamide/pilocarpine. Timolol alone did not cause substantial obstruction, which suggests that fixed combinations of PLMs had an increased risk of LDO (Kashkouli et al., 2008).

### 4.2 Prostaglandin analogs (PGAs)

#### 4.2.1 Skin pigmentation

Eyelid pigmentation (0%–26%) and eyelash bristles (0%–77%) represent a frequent periorbital manifestation associated with PGAs, with an increased frequency if used for >3 months (S et al., 2018) (Figure 3B). Inoue et al. reported there were no significant differences in the frequency (4%–6%) of eyelid pigmentation after >3 months of latanoprost, tafluprost, bimatoprost, travoprost, or isopropyl unoprostone use (Inoue et al., 2012a). Eyelash bristles, however, occurred significantly less with unoprostone (8%) compared with the other four drugs (26%–54%).

#### 4.2.2 Meibomian gland dysfunction (MGD)

**FIGURE 4:**
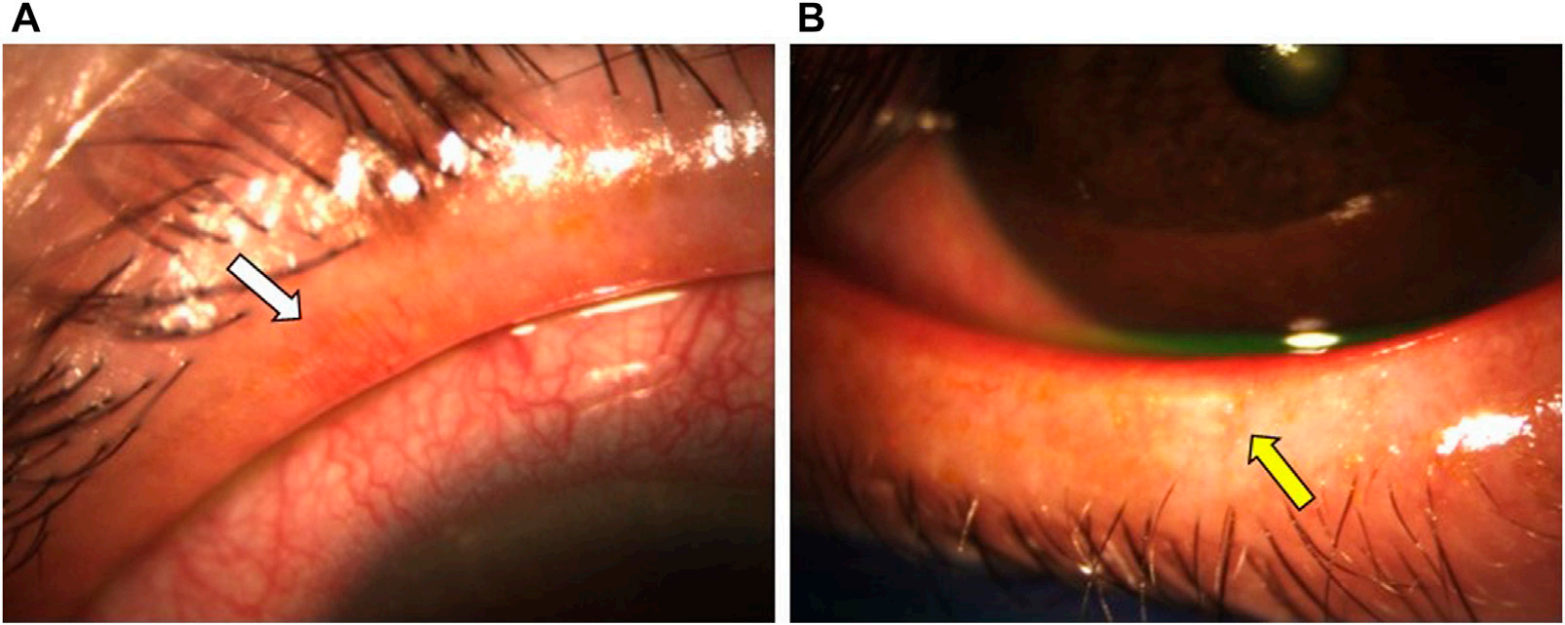
A 61-year-old female patient with a 7-year history of primary open-angle glaucoma (POAG) was treated with BAK-containing 0.005% latanoprost qHs eyedrops. **(A,B)** Significant lid margin erythema, telangiectasias (white arrow), meibomian gland clogging (yellow arrow). Footnote: Written informed consent was obtained from the patient to publish the clinical images.

**FIGURE 5:**
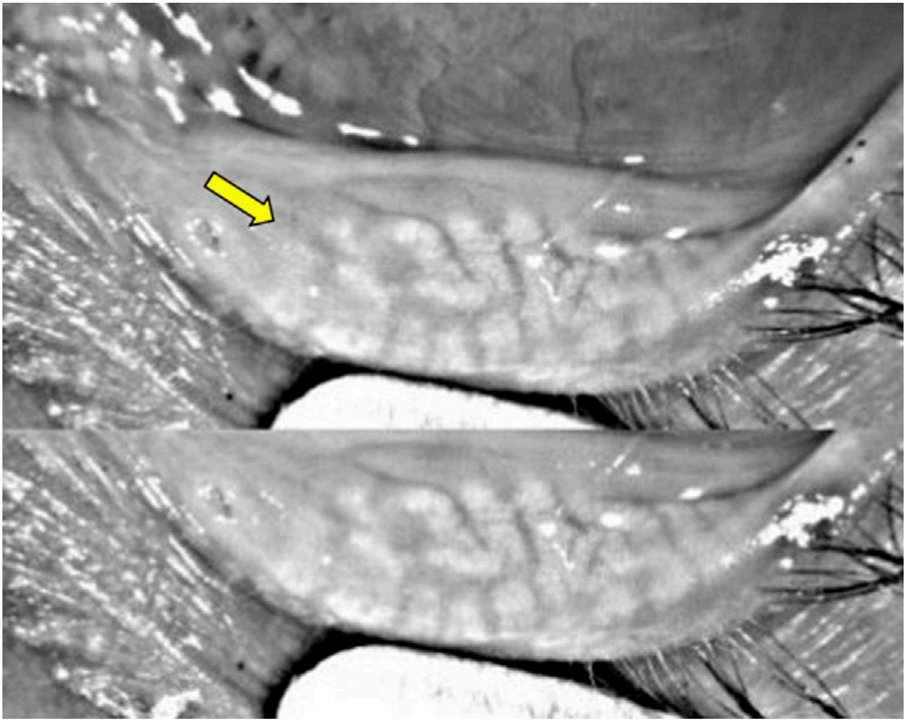
Keratograph analysis from a patient with a 12-year history of POAG treated with BAK-containing 0.005% latanoprost qHs eyedrops showing significant meibomian gland dropout (yellow arrow).

Mocan et al. reported a significantly higher prevalence of MGD in glaucoma patients managed with PGA monotherapy than those treated with other PLMs (92% vs 58%). The obstructive form of MGD was documented in 96% of patients from the PGA group (Mocan et al., 2016). Moreover, other ocular surface parameters, including the Ocular Surface Disease Index (OSDI), tear-film breakup time (TFBUT), lissamine green staining, and Schirmer scores were significantly worse in patients treated with PGA monotherapy compared to healthy controls (Mocan et al., 2016). The pathogenic mechanisms of MGD in patients treated with PGA remains poorly understood. Some authors suggest that subclinical inflammation of the conjunctiva results in MG dropout and dysfunction (Agnifili et al., 2018). Agnifili et al. reported a significant reduction in the mean acinar area (MAA) and density (MAD), which are respective surrogates of reduced meibum production and glandular dropout, and higher interstice inhomogeneity, which reflects MG and tarsal inflammation, in patients treated with PGAs (Agnifili et al., 2018). These findings were significantly higher in preservative PGA-treated patients than those managed with PF-PGAs. In another study, Arita et al. reported significantly higher lid margin abnormalities (i.e., vascular tortuosity, irregular lid margin, replacement of the mucocutaneous junction, and plugged MG orifices) (Figures 4A,B), which are associated with MGD and conjunctival inflammation, and higher Meibo-scores, implying increased MG dropout, in patients treated with PGA compared with β-blocker treated eyes and healthy controls (Arita et al., 2012) (Figure 5). The authors suggest that the lid margin abnormalities in glaucoma-treated eyes support the hypothesis that subclinical inflammation predates MG alterations (Arita et al., 2012). Recurrent inflammation resulting from prolonged exposure to PGA might lead to meibum stagnation with subsequent keratinization of MG orifices (i.e., obstructive MGD) (S et al., 2018).

#### 4.2.3 Conjunctival hyperemia

**FIGURE 6:**
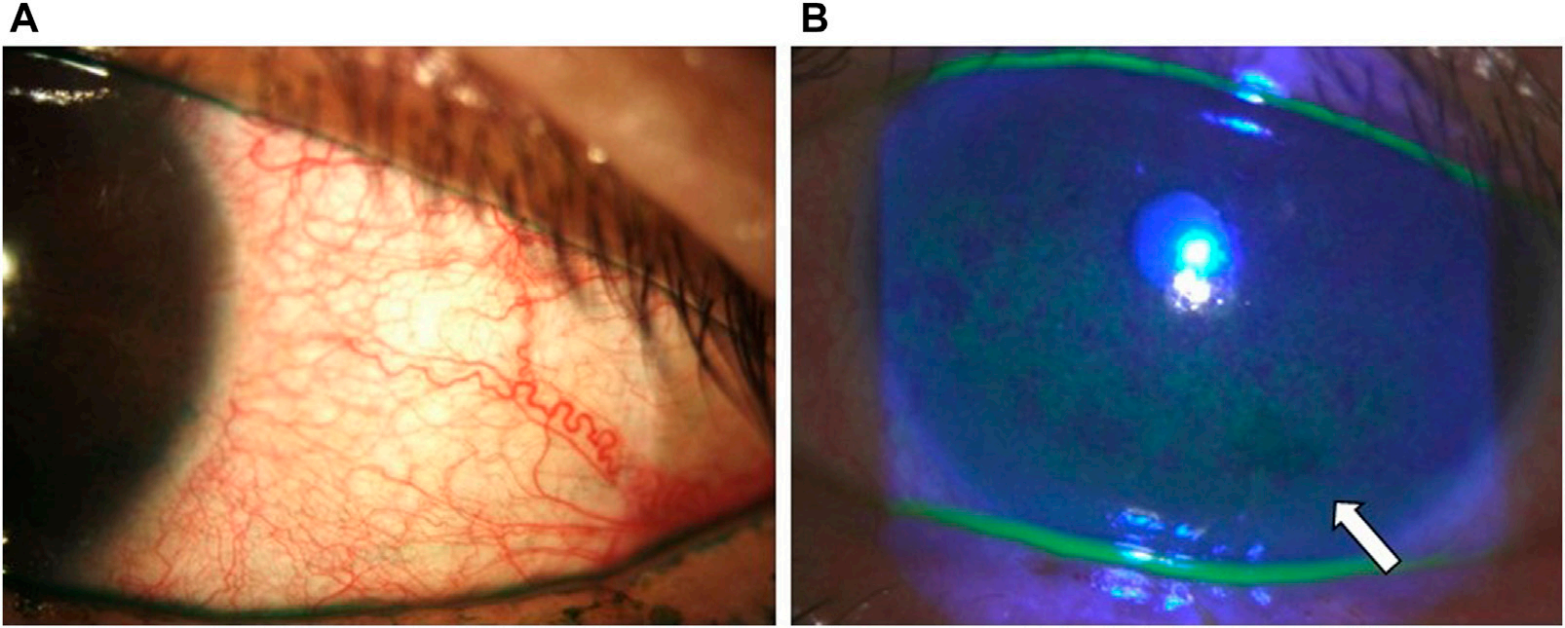
Clinical photographs of the patient from Figure 4 showing **(A)** conjunctival hyperemia with lissamine green staining and **(B)** central and inferior corneal staining (white arrow).

A recent meta-analysis performed by Tang et al. reported that the frequency of conjunctival hyperemia was significantly higher in bimatoprost (40%) compared with travoprost (39%) and latanoprost (28%) (Tang et al., 2019). Although unclear, PGAs are suspected of inducing the production of NO synthase, which may lead to conjunctival hyperemia due to their vasodilatory properties (Astin et al., 1994) (Figure 6A).

#### 4.2.4 Conjunctival goblet cell (GC) dropout

Human studies report GC loss after long-term treatment with BAK-containing PGA eyedrops and after short-term exposure to BAK alone (Pisella et al., 2004). On the other hand, Mastropasqua et al. described an increase in GC density after 6 months of therapy with PF-tafluprost. This finding can be explained by the ability of PG to stimulate the secretion and proliferation of mucin in numerous mucosal surfaces, including the conjunctiva (Mastropasqua et al., 2013). Interestingly, the authors report a transient increase in GC density after 1 month of preserved latanoprost. The GC density, however, returned to baseline after 6 months, suggesting that over time, the positive effect of the PG derivative is nullified by the preservative (Mastropasqua et al., 2013).

#### 4.2.5 Tear film

Using the Schirmer test I, Agnifili et al. compared tear production between healthy controls and groups of patients using several anti-glaucoma regimes, including combinations of preserved PGAs with timolol maleate and PF-bimatoprost and timolol (Agnifili et al., 2018). The tear production of healthy controls was significantly higher than in all therapy groups, including the fixed combination of PF-bimatoprost and timolol (18.4 ± 5.5 mm *vs*. 9.8 ± 3.5 mm). Interestingly, the unfixed combination of latanoprost and timolol yielded significantly worse Schirmer scores than the fixed combinations of timolol and different PGAs (latanoprost, travoprost, and bimatoprost) (Agnifili et al., 2018).

#### 4.2.6 Corneal thickness

A significant reduction in the central corneal thickness (CCT) was documented in human eyes after 8 weeks of treatment with either 0.03% bimatoprost, 0.004% travoprost, or 0.005% latanoprost compared with controls. No difference between PGAs was found (Hatanaka et al., 2009). Human studies have shown central corneal thinning after long-term exposure to PGAs, possibly due to increased activity of MMPs relative to TIMPs in the corneal epithelium and stroma (Lopilly Park et al., 2012). Upregulation of MMPs, mainly MMP-2 and -9, has been reported in stromal tissue after corneal ablative procedures and corneal erosions (Jadczyk-Sorek et al., 2022). In another study, Lopilly Park et al. did not find a significant reduction in the CCT of human eyes after 1 year of treatment with PGAs (Lopilly Park et al., 2012). In the same study, however, the authors reported that rabbit corneas exhibited corneal thinning caused by a decrease in collagen type 1 after PGA treatment. Also, a significant increase in MMP-1 and MMP-9 and a reduction in TIMP-1 were found in rabbit corneas (Lopilly Park et al., 2012). Differences in the collagen distribution between the human and rabbit corneas might also explain humans’ lack of significant corneal thinning. Table 5 presents the recent relevant *in-vivo, ex-vivo,* and *in-vitro* animal studies reporting OSD manifestations of preserved PLMs.

**TABLE 5:** Recent relevant *in-vivo, ex-vivo,* and *in-vitro* animal studies reporting ocular surface disease manifestations of PLMs.

	Cell type	Agents used	Study description	Preservatives	Findings
References					
Krauss et al. (2011)	*In vivo* OHT model in monkeys, dogs, and rabbits	NO-donating PGAs	To compare the hypotensive effects between LAT and NO-donating LAT	BAK	NO-donating LAT was more effective in lowering the intraocular pressure with no ocular side effects reported
Taketani et al. (2014)	*In vitro* mouse adipocytes	PGAs	Evaluate the effects of all PGAs on adipogenesis	None	All PGAs, except unoprostone, promoted lipolysis and suppressed adipogenesis, potentially explaining DUES
Jiang et al. (2022)	*Ex-vivo* mouse MGs	PGAs	Evaluate the effects of LAT on chemokine and cytokine secretion	None	LAT induced inflammation in Meibomian gland epithelial cells and suppressed differentiation by overexpressing of IL-1β, IL-6, TNF-α, MMP-9, among other cytokines
Young et al. (1990)	*Ex-vivo* rabbit conjunctiva	Miotics and BBs	To evaluate the effects of PIL and TIM on the conjunctiva	BAK	PIL produced higher myofibroblastic cell proliferation and thickened conjunctival epithelium and stroma
Pisella et al. (2000)	*In vivo* and *ex-vivo* rabbit cornea and conjunctiva	BBs	Evaluate the ocular surface tolerance to preserved and PF TIM	BAK	The PF-formulation exhibited significantly decreased TFBUT compared with BAK-containing TIM. Furthermore, PF-formulations also had less histological stromal edema
Liang et al. (2011)	*In vivo* rabbit cornea and conjunctiva	BBs and PGAs	Assess the toxicological profile of TRV/TIM and LAT/TIM fixed combinations	BAK, PQ	BAK-containing LAT/TIM produced more hyperemia, chemosis, tearing, and cytotoxicity (assessed by IVCM) compared to PQ-preserved TRV/TIM
Ayaki et al. (2012)	*In vitro* rabbit and bovine cornea	CAIs, PGAs, BBs, and AAs	To evaluate *in-vitro* cytotoxicity on corneal epithelial cells of BAK-containing PLMs	BAK	Decreased cell viability scores across most CAIs, B-blockers, and PGAs, or their fixed combinations
Shin et al. (2015)	*Ex-vivo* rat conjunctiva, cornea, and aqueous humor	AAs	Evaluate effect of brimonidine in the level of various inflammatory markers	PUR	Corneoconjunctival levels of inflammatory markers (TNFa, IL-1a, IL-1b, IL-6) were significantly lower in the brimonidine group, but increased in the aqueous humor
Lee et al. (2015)	*In vivo* rabbit cornea and conjunctiva	PGAs	Evaluate the ocular surface effects of various preserved PGAs	BAK, PQ	Decreased conjunctival goblet cell density, increased corneal pyknotic changes, and increased tear IL-6 were found in BAK-containing formulations
Kim et al. (2015)	*In vivo* mouse cornea	PGAs	Evaluate the effects of preserved and PF PGAs	BAK, PQ	PQ and PF formulations showed less SPK, epithelial desquamation, anisocytosis, and cell shrinkage compared with BAK
Lin et al. (2018)	*In vitro* rabbit, monkey, dog, pig, and human corneas	ROCK inhibitors	Preclinical characterization of netarsudil comparing its effects with other rho-associated protein kinase inhibitors	None	Netarsudil exhibited significant intraocular pressure reductions with only mild hyperemia
	*In vivo* rabbit conjunctiva				
Leary et al. (2021)	*In vivo* dog conjunctiva	ROCK inhibitors	Evaluate the safety and efficacy of netarsudil	BAK	Increased conjunctival hyperemia in treated eyes compared with balance salt solution

PLMs, pressure-lowering medications; NO, nitric oxide; PGAs, prostaglandin analogs; LAT, latanoprost; BAK, benzalkonium chloride; DUES, deepening upper eyelid sulcus; MGs, Meibomian glands; IL, interleukin; TNF, tumor necrosis factor; MMP, matrix metalloproteinase; BBs, beta blockers; PIL, pilocarpine; TIM, timolol; PF, preservative-free; TFBUT, tear film breakup time; TRV, travoprost; PQ, polyquad; IVCM, in-vivo confocal microscopy; CAIs, carbonic anhydrase inhibitors; AAs, alpha agonists; ROCK, rho kinase.

#### 4.2.7 Pseudodendritic keratitis (PSK)

PSK is uncommon in patients using PLMs. It is characterized by pseudodendritic central and lower corneal lesions surrounded by SPK (Chang et al., 2021). A recent retrospective case series reported 31 events (19 patients) of PSK, 52% of them associated with PGAs and 97% to BAK-containing PLMs (Chang et al., 2021). Management includes using therapeutic soft contact lens, lubricant eyedrops, andto discontinuation, decrease, or change of the PLM used (Chang et al., 2021). Due to their similarities, PSK could get confused with herpetic simplex keratitis. However, the latter is characterized by dendrites with central fluorescein staining and terminal bulbs that can be found anywhere in the cornea (Chang et al., 2021).

### 4.3 Carbonic anhydrase inhibitors (CAIs)

#### 4.3.1 Contact dermatitis

Delaney et al. reported periocular contact dermatitis in 14 patients after 20.4 weeks of initiating BAK-containing dorzolamide. Of those, 13 patients were using preserved β-blockers for 34.2 months (Delaney et al., 2002). Dermatitis wholly resolved in 8 cases (57%) after discontinuing dorzolamide, and in the remaining 6 (43%), resolution occurred after the topical β-blocker was also stopped. Negative patch testing to β-blockers, BAK, and dorzolamide was reported in all cases. The authors hypothesized that a false-negative reaction, or simply irritation rather than sensitization, might have occurred (Delaney et al., 2002).

#### 4.3.2 Drug-induced ectropion

Hegde et al. reported 13 patients who developed drug-induced ectropion after exposure to dorzolamide (7 cases, 53%), brimonidine (3 cases, 23%), and other agents, including β-blockers, latanoprost, and preserved lubricant eyedrops (Hegde et al., 2007). This effect was reversible after drug discontinuation and a short course of topical steroids in 9 cases. However, the remaining patients who did not receive steroids were successfully managed with surgical correction (Hegde et al., 2007).

#### 4.3.3 Meibomian gland dysfunction (MGD)

Han et al. reported that, compared with the therapeutic concentration (50 µg/mL) of dorzolamide, a 10-fold higher concentration of 500 µg/mL significantly reduces iHMGEC proliferation while increasing iHMGEC differentiation (Han et al., 2020). The authors suggest that dorzolamide might enhance the expression of hypoxia-inducible factor (HIF)-1α, which facilitates iHMGEC differentiation by triggering a cellular response to hypoxic stress. However, the therapeutic concentration of dorzolamide did not elicit such an effect (Han et al., 2020).

#### 4.3.4 Conjunctival hyperemia

he incidence of hyperemia in dorzolamide users ranges from 7% to 21% (Adamsons et al., 1998; Ott et al., 2005); however, brinzolamide, another CAI, has a lower prevalence (<3%) of hyperemia due to its physiological pH, which enhances tolerability and compliance (Lusthaus and Goldberg, 2017).

### 4.4 α-Adrenergic agonists

#### 4.4.1 Meibomian gland dysfunction (MGD)

Brimonidine triggers the upregulation of sterol regulatory element-binding protein (SREBP)-1, a lipogenesis regulator that synthesizes lipid, cholesterol, and fatty acid production enzymes. Moreover, brimonidine enhances the conversion of SREBP-1 to its mature form, thus promoting lipid accumulation and differentiation iHMGECs (Han et al., 2018). The authors conclude that dry eye symptoms associated with brimonidine, including blurry vision, ocular discomfort, and DED, might be related to corneal toxicity, GC loss, and conjunctival inflammation rather than MG dropout (Han et al., 2020).

#### 4.4.2 Conjunctival allergy

Allergic reactions are another frequent side effect of alpha agonists. Although unclear, the pathogenic mechanism could be related to a volume decrease and subsequent widening of the intracellular spaces between conjunctival cells, leading to an entry portal for external allergens (Yeh et al., 2021). Butler et al. reported an incidence of allergic reaction in 48% of apraclonidine users (Araie et al., 2015). This side effect was more common in women and led to drug discontinuation after an average latency of 4.7 months (Butler et al., 1995). In the case of brimonidine, the incidence of allergy ranges from 4.7% to 25%, occurring at a mean time interval of 6–9 months (Yeh et al., 2021). The prevalence of hyperemia is 13% and 8% for apraclonidine and brimonidine users, respectively (Robin et al., 1995; Mundorf et al., 2003).

### 4.5 Cholinergic agents

#### 4.5.1 Contact dermatitis

Allergic contact dermatitis has been previously reported in topical pilocarpine users. O’Donnell and Foulds presented a patient with negative patch testing to topical PLM ingredients at days 4 and 7 (O'Donnell and Foulds, 1993). However, using the prick-testing method with unpreserved pilocarpine elicited a 10-mm wheal after 30 min, a finding suggestive of contact urticaria. In a similar fashion, Cusano et al. described of a patient with 1-year history of eyelid dermatitis and negative patch testing, but who developed an inflammatory reaction after repeated open application Test with pilocarpine eye drops (Cusano et al., 1993).

#### 4.5.2 Meibomian gland dysfunction (MGD)

Zhang et al. reported significant cell atrophy and death of cultured immortalized human MG epithelial cells (iHMGEC) with 0.04% pilocarpine, a tenfold lower than the clinical dose (Zhang et al., 2017). The standard 0.4% pilocarpine-induced impaired cellular adherence, perinuclear vesicle accumulation, which heralds cell death, and decreased survival of iHMGEC. Although elusive, pilocarpine-induced MG dropout might be related to its effects on muscarinic acetylcholine receptor 3, present in iHMGEC (Wu et al., 2020).

### 4.6 Rho-kinase (ROCK) inhibitors

#### 4.6.1 Postoperative eyelid wound dehiscence

It is defined as a break or split in the eyelid after a previously closed surgical incision site (Sandy-Hodgetts et al., 2015). Kim et al. reported the case of an 81-year-old male with glaucoma managed with PLMs, including 0.02% netarsudil. The patient underwent upper eyelid Mohs surgery and lid repair due to basal cell carcinoma. Interestingly, the patient suffered three episodes of wound dehiscence, requiring repair in two (Kim et al., 2021). After the last episode, the patient discontinued the netarsudil eyedrops, and 2 weeks later, he developed granulation tissue, and the wound healed appropriately. In diabetic foot ulcer rat models, overexpression of ROCK1 has been shown to increase wound healing (Wang et al., 2022). Inhibition of MLC-phosphatases by the ROCK pathway leads to long-lasting contraction of myofibroblasts, which is required for wound healing (Saha et al., 2022). Thus, ROCK inhibition with netarsudil could lead to poor wound healing.

#### 4.6.2 Conjunctival hyperemia

The Rho Kinase Elevated IOP Treatment (ROCKET) Trials reported an incidence of conjunctival hyperemia of 50%–53% and 59% in eyes receiving 0.02% netarsudil once and twice daily, respectively. These results were significantly higher than the 8%–11% incidence of conjunctival hyperemia in eyes receiving 0.5% timolol twice daily (Serle et al., 2018). In a rabbit model, Watabe and coworkers demonstrated that ROCK inhibitors caused vasodilation of the ciliary arteries due to a calcium-independent mechanism. This contrasts with the PLMs tafluprost, a PGA, and levobunolol, a β-blocker, which cause relaxation of the ciliary arteries by decreasing calcium concentration in the intracellular space (Watabe et al., 2011). As stated above, ROCKs contract the trabecular meshwork by phosphorylation of MLCs. Thus, conjunctival hyperemia associated with ROCK inhibitors could be related to vasodilation of ciliary arteries due to smooth muscle relaxation secondary to MLC phosphorylation (Watabe et al., 2011).

#### 4.6.3 Subconjunctival hemorrhage

Singh et al. reported a higher incidence of subconjunctival hemorrhage in patients managed with once-a-day 0.02% netarsudil eyedrops compared with twice-a-day 0.5% timolol (17% vs 2%). Among patients managed with netarsudil, the hemorrhage was mild in 92% of cases, self-limiting in 96%, and requiring drug discontinuation in 1% (Singh et al., 2020).

#### 4.6.4 Drug-induced cicatrizing conjunctivitis (DICC)

Meirick et al. reported 16 patients who developed reversible punctum stenosis after an average time of 14 months of 0.02% netarsudil use. Of those, 13 (81%) patients had symptomatic epiphora, leading to drug discontinuation in 7 cases. Histopathological analysis from the conjunctiva and punctum of one patient showed nonspecific lymphocytic inflammation without eosinophils (Meirick et al., 2022). This contrasts with the findings encountered in eyes with the non-reversible scarring inflammation in DICC, which is typically associated with β-blockers (Singh et al., 2022). Punctal scarring has not been reported with ripasudil, another ROCK inhibitor. Compared with ripasudil, netarsudil has a NET inhibitor function. However, the effect of NET inhibition and punctum scarring remains unknown (Meirick et al., 2022).

#### 4.6.5 Corneal edema

Wisely et al. reported five patients who developed reticular bullous corneal epithelial edema after a mean time of 5.4 (range: 2–8) weeks of netarsudil use (Wisely et al., 2020). Four patients had a prior history of corneal edema due to different causes. The remaining patient had a previous history of anterior uveitis, which predisposes to corneal edema. In all cases, the epithelial edema resolved after a mean time of 7.4 (range: 2–12) weeks of discontinuing netarsudil (Wisely et al., 2020).

ROCKs and tight junctions, including zonula occludins (ZO)-1, oversee osmotic regulation in epithelial surfaces (Chmiel and Gardel, 2022). ROCK inhibitors could lead to epithelial edema by increasing the permeability of tight junctions, thus allowing fluid to percolate from the corneal stroma into the epithelium (Chmiel and Gardel, 2022; Lyons et al., 2022). Corneas with a prior history of developing corneal edema might be more susceptible to ROCK inhibition (Wisely et al., 2020). However, the latter remains unknown.

### 4.7 Nitric oxide (NO)-donating prostaglandin analogs (PGAs)

#### 4.7.1 Conjunctival hyperemia

The prevalence of hyperemia associated with latanoprostene bunod ranges from 2% to 18% (Lo et al., 2022). Besides the NO synthase induced vasodilation (See Section 4.1.3), another potential mechanism of conjunctival hyperemia could be related to the pro-inflammatory properties of the prostaglandin F2α molecule itself (Kawase et al., 2016).

#### 4.7.2 Superficial punctate keratitis (SPK)

In an open-label clinical study of healthy subjects, Araie et al. reported a prevalence of 54.2% of SPK among healthy users of once-a-day 0.024% latanoprostene bunod. However, in all cases, the SPK was mild and clinically insignificant (Araie et al., 2015). In a recent meta-analysis of randomized controlled trials (RCTs), the prevalence of SPK ranged from 1.1% to 4.3% (Lo et al., 2022).

## 5 Preservatives

Preservative agents, including BAK, polyquaternium-1 (Polyquad), Sofzia^®^, and Purite^®^, influence corneal penetration of the active substance through their surface wetting properties and provide bacteriostatic activity (Servat and Bernardino, 2011). Within the eye, the lipophilic nature of most preservatives renders immediate binding to ocular tissues after application. However, many animal and human studies have shown that preservatives are culprits of inducing or worsening ophthalmic formulations’ toxic, allergic, and inflammatory reactions, including PLMs (Kim et al., 2015; Muz et al., 2021).

BAK is the most frequently used preservative in ophthalmic preparations (Muz et al., 2021). It is commonly used as a cationic surfactant, a phase transfer agent in the chemical industry, and a biocidal agent due to its activity against fungi and Gram-positive and Gram-negative bacteria (Muz et al., 2021). However, BAK-preserved formulations have been shown to trigger dose-dependent inflammation, tear instability, increased osmolarity, corneal and conjunctival epithelial cytotoxicity, squamous metaplasia, and GC loss (Kim et al., 2015). To reduce the toxicity of BAK-preserved formulations, less toxic formulations were designed, including Sofzia^®^, Polyquad, and Purite^®^. However, PF antiglaucoma formulations are available and should be considered first-line, mainly in patients with preexisting OSD.

Damage to the limbal stem cells (LSCs) and corneal epitheliopathy has been associated with multiple PLMs. However, studies suggest that preservatives, rather than the active ingredient, have been identified as culprits. LSCs, which have a high proliferation, differentiation, and migration capacity, reside in the corneoscleral limbus (Guclu et al., 2021). LSCD results from an impaired function and reduced number of LSCs which, in turn, can lead to corneal conjunctivalization, persistent epithelial defects (PEDs), scarring, and vision loss. The concept of “iatrogenic LSCD” in eyes managed with PLMs was first coined by Schwartz and Holland (Schwartz and Holland, 2001). Güçlü et al. evaluated the limbal epithelium thickness (LET) in patients treated with either one, two, three, or four-drug regimens of BAK-containing anti-glaucoma medications (Guclu et al., 2021). The authors found no difference in the LET between treated groups; however, it was significantly lower compared to non-treated healthy eyes (64.1 ± 9.1 µm *vs*. 76.0 ± 11.5 µm). Moreover, there was a positive correlation between increased LET and increased Schirmer (*r* = 0.4), TFBUT (*r* = 0.37), and central corneal epithelial thickness (CCET; *r* = 0.37) (Guclu et al., 2021). A decreased significant LET is also reported in DED patients (Francoz et al., 2011). PLMs might decrease LET due to increased epithelial turnover or chronic inflammation (Francoz et al., 2011). On the other hand, the association between decreased LET and decreased CCET may result from decreased proliferation, differentiation, and migration of reduced LSCs (Guclu et al., 2021).

A morphologic IVCM study by Mastropasqua et al. analyzed the density of dendritic cells (DCs) and the regularity of the limbal transition epithelium (LTE) in eyes treated with single, double, and triple or more anti-glaucoma eyedrops regimes (Mastropasqua et al., 2015). Eyes managed with preserved β-blockers and preserved PGAs exhibited higher DCs density and worsened LTE irregularity compared with PF-drugs. A higher DC density results from BAK-induced local immune system activation, whereas the LTE irregularity, observed as punctate reflecting elements with IVCM analysis, represents an additional sign of inflammation (Mastropasqua et al., 2015). Moreover, eyes treated with preserved drugs significantly increased HLA-DR and IL-6 compared with PF drugs. This supports the theory that inflammation might be the initial cascade step leading to limbal abnormalities (Mastropasqua et al., 2015).

Superficial punctate keratitis (SPK) encircles a group of corneal epithelial lesions with varying morphology and can be observed as corneal staining at the slit-lamp. The prevalence of SPK in anti-glaucoma eyedrop users is reported to be as high as 70% (Lajmi et al., 2021) (Figure 6B). Using *in vivo* confocal microscopy (IVCM), Mastropasqua et al. reported that the central corneal DC density significantly increased in patients with preserved compared to those receiving PF PGAs and β-blockers (Mastropasqua et al., 2016). Additionally, the corneal DC density significantly correlated with corneal staining and OSDI scores (*p* < 0.001). These results resemble those found in the limbal epithelium, suggesting an increased inflammatory response in the corneal epithelium with a subsequent increase in signs and symptoms (Mastropasqua et al., 2016). Ye et al. reported a significant association between increased fluorescein staining and epithelial thickness in the central and paracentral cornea, indicating that abnormal staining might predict corneal epithelial thinning (Ye et al., 2022). SPK is also reported in 5%–10% of patients using netarsudil and in 1%–4% of latanoprostene bunod users (Batra et al., 2021; Lo et al., 2022).

## 6 Ocular surface disease and quality of life in glaucoma patients

Quality of life (QoL) refers to patients’ perception of their daily wellbeing (Kumar et al., 2020). Unfortunately, QoL can be severely affected in patients with glaucoma and OSD, which often coexist (Camp et al., 2015; Tirpack et al., 2019). Studies report a significant association between the increased number of PLMs and decreased emotional wellbeing scores, with African American patients experiencing worse QoL scores (Camp et al., 2015). Abegão Pinto et al. prospectively evaluated the change in visual-related QoL, assessed by the Glaucoma Symptom Scale (GSS), in patients with glaucoma after switching from preserved IOP-lowering therapy to PF-timolol/dorzolamide fixed combination (TDFC). After 8 weeks of treatment with PF-TDFC, there was a significant improvement in the GSS-related symptom, function, and total scores (Abegao Pinto et al., 2014). Accordingly, Kumar et al. found significantly worse QoL scores between patients using BAK-containing travoprost and the PF-travoprost and control groups (Kumar et al., 2020). Interestingly, there was no difference between the reported QoL in the PF-travoprost and control groups, suggesting the harmful role of preservatives in OSD and QoL in glaucoma patients (Kumar et al., 2020).

## 7 Future directions

OSD symptoms are detrimental to glaucoma patients’ perceived QoL, compliance to therapy, reliability of diagnostic tests, poor surgical outcomes, and disease progression and visual outcomes (Zhang et al., 2019). Thus, addressing OSD in glaucoma is crucial. A survey-based study reported that 100% of Canadian glaucoma specialists agreed that a good approach to OSD in patients might improve QoL, whereas 97% agreed that it could result in enhanced glaucoma outcomes (Muzychuk et al., 2020). Furthermore, only 22% agreed that OSD is currently being managed appropriately, and 92% agreed that a stepwise approach should be undertaken to address OSD in glaucoma. Accordingly, the authors proposed an algorithm consisting of 1) optimizing topical glaucoma medications by using combined and PF formulations; 2) promoting ocular surface health (i.e., PF-lubricants and gels, omega-3 fatty acid supplementation); 3) enhancing OSD therapy with steroids immunomodulatory drugs (i.e., cyclosporine A, autologous serum); and 4) considering surgical interventions (Muzychuk et al., 2020). In this regard, minimally invasive glaucoma surgery and slow delivery systems such as the bimatoprost implant have emerged as a possible solution for reducing IOP with fewer ocular surface adverse effects compared with traditional surgical and non-surgical IOP-lowering methods (Schoenberg et al., 2015; Schweitzer et al., 2020; Shirley, 2020). A recent prospective cohort study reported a significant reduction in the number of PLMs, a substantial improvement in OSDI and TFBUT scores, and conjunctival hyperemia in patients who underwent combined cataract surgery with trabecular micro-bypass stent(s) implantation (Schweitzer et al., 2020). More randomized prospective trials with extensive follow-up are required to assess the direct impact of MIGS on the ocular surface.

## 8 Conclusion

OSD is a frequently overlooked condition resulting from glaucoma therapy, with age, use of BAK-containing and multiple anti-glaucoma medications, concomitant systemic comorbidities, and previous DED as the most frequent associated risk factors. Eye care specialists must remain aware of the adverse effects of PLMs and, thus, actively inquire about ocular surface symptoms. Upon diagnosis of OSD, a severity-based, stepladder approach consisting of optimizing glaucoma treatment by switching to fixed and PF combinations, using PF ocular lubricants, prescribing short courses of topical steroids with or without immunomodulatory therapy, and considering early surgical intervention are required to enhance medication adherence and improve glaucoma outcomes.
